# The Social Consequences of Poverty: An Empirical Test on Longitudinal Data

**DOI:** 10.1007/s11205-015-0983-9

**Published:** 2015-05-17

**Authors:** Carina Mood, Jan O. Jonsson

**Affiliations:** Institute for Futures Studies, Box 591, 101 31 Stockholm, Sweden; Swedish Institute for Social Research (SOFI), Stockholm University, Stockholm, Sweden; Nuffield College, OX1 1NF Oxford, England, UK

**Keywords:** Poverty consequences, Material deprivation, Poverty definitions, Social relations, Sweden

## Abstract

Poverty is commonly defined as a lack of economic resources that has negative social consequences, but surprisingly little is known about the importance of economic hardship for social outcomes. This article offers an empirical investigation into this issue. We apply panel data methods on longitudinal data from the Swedish Level-of-Living Survey 2000 and 2010 (n = 3089) to study whether poverty affects four social outcomes—close social relations (social support), other social relations (friends and relatives), political participation, and activity in organizations. We also compare these effects across five different poverty indicators. Our main conclusion is that poverty in general has negative effects on social life. It has more harmful effects for relations with friends and relatives than for social support; and more for political participation than organizational activity. The poverty indicator that shows the greatest impact is material deprivation (lack of cash margin), while the most prevalent poverty indicators—absolute income poverty, and especially relative income poverty—appear to have the least effect on social outcomes.

## Introduction

According to the most influential definitions, poverty is seen as a lack of economic resources that have negative social consequences—this is in fact a view that dominates current theories of poverty (Townsend 1979; Sen 1983; UN 1995), and also has a long heritage (Smith 1776/1976). The idea is that even when people have food, clothes, and shelter, economic problems lead to a deterioration of social relations and participation. Being poor is about not being able to partake in society on equal terms with others, and therefore in the long run being excluded by fellow citizens or withdrawing from social and civic life because of a lack of economic resources, typically in combination with the concomitant shame of not being able to live a life like them (e.g., Sen 1983). Economic hardship affects the standard of life, consumption patterns, and leisure time activities, and this is directly or indirectly related to the possibility of making or maintaining friends or acquaintances: poverty is revealed by not having appropriate clothes, or a car; by not being able to afford vacation trips, visits to the restaurant, or hosting dinner parties (e.g., Mack and Lansley 1985; Callan et al. 1993)—in short, low incomes prevent the poor from living a life in “decency” (Galbraith 1958).

The relational nature of poverty is also central to the social exclusion literature, which puts poverty in a larger perspective of multiple disadvantages and their interrelationships (Hills et al. 2002, Rodgers et al. 1995; Room 1995). While there are different definitions of the social exclusion concept, the literature is characterized by a move from distributional to relational concerns (Gore 1995) and by an emphasis on the importance of social integration and active participation in public life. The inability of living a decent or “ordinary” social life may in this perspective erode social networks, social relations, and social participation, potentially setting off a downward spiral of misfortune (Paugam 1995) reinforcing disadvantages in several domains of life. This perspective on poverty and social exclusion is essentially sociological: the playing field of the private economy is social. It is ultimately about individuals’ relations with other people—not only primary social relations, with kin and friends, but extending to secondary relations reflected by participation in the wider community, such as in organizations and in political life (UN 1995).

Despite the fact that the social consequences of limited economic resources are central to modern perspectives on poverty and marginalization, this relation is surprisingly seldom studied empirically. Qualitative research on the poor give interesting examples on how the negative effects of poverty works, and portray the way that economic problems are transformed into social ones (Ridge and Millar 2011; Attree 2006). Such studies, however, have too small sample sizes to generalize to the population, and they cannot tell us much about the range of the problem. The (relatively few) studies that have addressed the association between poverty and social outcomes on larger scale tend to verify that the poor have worse social relations (Böhnke 2008; Jonsson and Östberg 2004; Levitas 2006), but Barnes et al. (2002) did not find any noteworthy association between poverty (measured as relative income poverty, using the 60 %-limit) and social relations or social isolation. Dahl et al. (2008) found no relation between poverty and friendships, but report less participation in civic organizations among the poor. All these studies have however been limited to cross-sectional data or hampered by methodological shortcomings, and therefore have not been able to address the separation of selection effects from potentially causal ones.

Our aim in this study is to make good these omissions. We use longitudinal data from the Swedish Level of Living Surveys (LNU) 2000 and 2010 to study how falling into poverty, or rising from it, is associated with outcomes in terms of primary and secondary social relations, including participation in civil society. These panel data make it possible to generalize the results to the Swedish adult population (19–65 in 2000; 29–75 in 2010), to address the issue of causality, and to estimate how strong the relation between economic vulnerability and social outcomes is. Because the data provide us with the possibility of measuring poverty in several ways, we are also able to address the question using different—alternative or complementary—indicators. Poverty is measured as economic deprivation (lack of cash margin, self-reported economic problems), income poverty (absolute and relative), and long-term poverty, respectively. The primary, or core, social outcomes are indicated by having social support if needed, and by social relations with friends and relatives. We expand our analysis to secondary, or fringe, social outcomes in terms of participation in social life at large, such as in civil society: our indicators here include the participation in organizations and in political life.

## Different Dimensions/Definitions of Poverty

In modern welfare states, the normal take on the issue of poverty is to regard it as the relative lack of economic resources, that is, to define the poor in relation to their fellow citizens in the same country at the same time. Three approaches dominate the scholarly literature today. The first takes as a point of departure the income deemed necessary for living a life on par with others, or that makes possible an “acceptable” living standard—defined as the goods and services judged necessary, often on the basis of consumer or household budget studies. This usage of a poverty threshold is often (somewhat confusingly) called *absolute income poverty*, and is most common in North America (cf. Corak 2006 for a review), although most countries have poverty lines defined for different kinds of social benefits. In Europe and in the OECD, the convention is instead to use versions of *relative income poverty*, defining as poor those whose incomes fall well behind the median income in the country in question (European Union using 60 % and OECD 50 % of the median as the threshold). As an alternative to using purchasing power (as in the “absolute” measure), this relative measure defines poverty by income inequality in the bottom half of the income distribution (Atkinson et al. 2002; OECD 2008).

The third approach argues that income measures are too indirect; poverty should instead be indicated directly by the lack of consumer products and services that are necessary for an acceptable living standard (Mack and Lansley 1985; Ringen 1988; Townsend 1979). This approach often involves listing a number of possessions and conditions, such as having a car, washing machine, modern kitchen; and being able to dine out sometimes, to have the home adequately heated and mended, to have sufficient insurances, and so on. An elaborate version includes information on what people in general see as necessities, what is often termed “consensual” poverty (e.g., Mack and Lansley 1985; Gordon et al. 2000; Halleröd 1995; van den Bosch 2001). Other direct indicators include the ability to cover unforeseen costs (cash margin) and subjective definitions of poverty (e.g., van den Bosch 2001). The direct approach to poverty has gained in popularity and measures of economic/material deprivation and consensual poverty are used in several recent and contemporary comparative surveys such as ECHP (Whelan et al. 2003) and EU-SILC (e.g., UNICEF 2012; Nolan and Whelan 2011).

It is often pointed out that, due to the often quite volatile income careers of households, the majority of poverty episodes are short term and the group that is identified as poor in the cross-section therefore tends to be rather diluted (Bane and Ellwood 1986; Duncan et al. 1993). Those who suffer most from the downsides of poverty are, it could be argued, instead the long-term, persistent, or chronically poor, and there is empirical evidence that those who experience more years in poverty also are more deprived of a “common lifestyle” (Whelan et al. 2003). Poverty persistence has been defined in several ways, such as having spent a given number of years below a poverty threshold, or having an average income over a number of years that falls under the poverty line (e.g., Duncan and Rodgers 1991; Rodgers and Rodgers 1993). The persistently poor can only be detected with any precision in longitudinal studies, and typically on the basis of low incomes, as data covering repeated measures of material deprivation are uncommon.

For the purposes of this study, it is not essential to nominate the best or most appropriate poverty measure. The measures outlined above, while each having some disadvantage, all provide plausible theoretical grounds for predicting negative social outcomes. Low incomes, either in “absolute” or relative terms, may inhibit social activities and participation because these are costly (e.g., having decent housing, needing a car, paying membership fees, entrance tickets, or new clothes). Economic deprivation, often indicated by items or habits that are directly relevant to social life, is also a valid representation of a lack of resources. Lastly, to be in long-term poverty is no doubt a worse condition than being in shorter-term poverty.

It is worth underlining that we see different measures of poverty as relevant indicators despite the fact that the overlap between them often is surprisingly small (Bradshaw and Finch 2003). The lack of overlap is not necessarily a problem, as different people may have different configurations of economic problems but share in common many of the experiences of poverty—experiences, we argue, that are (in theory at least) all likely to lead to adverse social outcomes. Whether this is the case or not is one of the questions that we address, but if previous studies on child poverty are of any guidance, different definitions of poverty may show surprisingly similar associations with a number of outcomes (Jonsson and Östberg 2004).

## What are the Likely Social Consequences of Poverty?

We have concluded that poverty is, according to most influential poverty definitions, manifested in the social sphere. This connects with the idea of Veblen (1899) of the relation between consumption and social status. What you buy and consume—clothes, furniture, vacation trips—in part define who you are, which group you aspire to belong to, and what view others will have of you. Inclusion into and exclusion from status groups and social circles are, in this view, dependent on economic resources as reflected in consumption patterns. While Veblen was mostly concerned about the rich and their conspicuous consumption, it is not difficult to transfer these ideas to the less fortunate: the poor are under risk of exclusion, of losing their social status and identity, and perhaps also, therefore, their friends. It is however likely that this is a process that differs according to outcome, with an unknown time-lag.

If, as outlined above, we can speak of primary and secondary social consequences, the former should include socializing with friends, but also more intimate relations. Our conjecture is that the closer the relation, the less affected is it by poverty, simply because intimate social bonds are characterized by more unconditional personal relations, typically not requiring costs to uphold.

When it comes to the secondary social consequences, we move outside the realm of closer interpersonal relations to acquaintances and the wider social network, and to the (sometimes relatively anonymous) participation in civil or political life. This dimension of poverty lies at the heart of the social exclusion perspective, which strongly emphasizes the broader issues of societal participation and civic engagement, vital to democratic societies. It is also reflected in the United Nation’s definition, following the Copenhagen summit in 1995, where “overall poverty” in addition to lack of economic resources is said to be “…characterized by lack of participation in decision-making and in civil, social, and cultural life” (UN 1995, p. 57). Poverty may bring about secondary social consequences because such participation is costly—as in the examples of travel, need for special equipment, or membership fees—but also because of psychological mechanisms, such as lowered self-esteem triggering disbelief in civic and political activities, and a general passivity leading to decreased organizational and social activities overall. If processes like these exist there is a risk of a “downward spiral of social exclusion” where unemployment leads to poverty and social isolation, which in turn reduce the chances of re-gaining a footing in the labour market (Paugam 1995).

What theories of poverty and social exclusion postulate is, in conclusion, that both what we have called primary and secondary social relations will be negatively affected by economic hardship—the latter supposedly more than the former. Our strategy in the following is to test this basic hypothesis by applying multivariate panel-data analyses on longitudinal data. In this way, we believe that we can come further than previous studies towards estimating causal effects, although, as is the case in social sciences, the causal relation must remain preliminary due to the nature of observational data.

## Data and Definitions

We use the two most recent waves of the Swedish Level-of-living Survey, conducted in 2000 and 2010 on random (1/1000) samples of adult Swedes, aged 18–75.^1^ The attrition rate is low, with 84 % of panel respondents remaining from 2000 to 2010. This is one of the few data sets from which we can get over-time measures of both poverty and social outcomes for a panel that is representative of the adult population (at the first time point, t_0_)—in addition, there is annual income information from register data between the waves. The panel feature obviously restricts the age-groups slightly (ages 19–65 in 2000; 29–75 in 2010), the final number of analyzed cases being between 2995 and 3144, depending on the number of missing cases on the respective poverty measure and social outcome variable. For ease of interpretation and comparison of effect sizes, we have constructed all social outcome variables and poverty variables to be dichotomous (0/1).^2^

In constructing poverty variables, we must balance theoretical validity with the need to have group sizes large enough for statistical analysis. For example, we expand the absolute poverty measure to include those who received social assistance any time during the year. As social assistance recipients receive this benefit based on having an income below a poverty line that is similar to the one we use, this seems justifiable. In other cases, however, group sizes are small but we find no theoretically reasonable way of making the variables more inclusive, meaning that some analyses cannot be carried out in full detail.

Our income poverty measures are based on register data and are thus free from recall error or misreporting, but—as the proponents of deprivation measures point out—income poverty measures are indirect measures of hardship. The deprivation measure is more direct, but self-reporting always carries a risk of subjectivity in the assessment. To the extent that changes in one’s judgment of the economic situation depend on changes in non-economic factors that are also related to social relations, the deprivation measure will give upwardly biased estimates.^3^ As there is no general agreement about whether income or deprivation definitions are superior, our use of several definitions is a strength because the results will give an overall picture that is not sensitive to potential limitations in any one measure. In addition, we are able to see whether results vary systematically across commonly used definitions.

### Poverty Measures

- *Economic deprivation* combines information from two survey questions:
  1. *Cash margin* whether the respondent can raise a given sum of money in a week, if necessary (in 2000, the sum was 12,000 SEK; in 2010, 14,000 SEK, the latter sum corresponding to approximately 1600 Euro, 2200 USD, or 1400 GBP in 2013 currency rates). For those who answer in the affirmative, there is a follow-up question of how this can be done: by (a) own/household resources, (b) borrowing.
  2. *Economic crisis* Those who claim that they have had problems meeting costs for rent, food, bills, etc. during the last 12 months (responded “yes” to a yes/no alternative).
  As economically deprived we classify those who (1) have no cash margin, or (2) can raise money only by borrowing *in combination with* having reported economic crisis.
- *Absolute poverty* is defined as either (a) having a disposable family income below a poverty threshold or (b) receiving social assistance, both assessed in 1999 (for the survey 2000) or 2009 (for the survey 2010). The poverty line varies by family type/composition according to a commonly used calculation of household necessities (Jansson 2000). This “basket” of goods and services is intended to define an acceptable living standard, and was originally constructed for calculating an income threshold for social assistance, with addition of estimated costs for housing and transport. The threshold is adjusted for changes in the Consumer Price Index, using 2010 as the base year. In order to get analyzable group sizes, we classify anyone with an income below 1.25 times this threshold as poor. Self-employed are excluded because their nominal incomes are often a poor indicator of their economic standard.
- *Deprived and income poor* A combination of the indicator of economic deprivation and the indicator of absolute poverty. The poor are defined as those who are economically deprived and in addition are either absolute income-poor or have had social assistance some time during the last calendar year.
- *Long*-*term poor* are defined as those interviewed in 2010 (2000) who had an equivalized disposable income that fell below the 1.25 absolute poverty threshold (excluding self-employed) or who received social assistance in 2009 (1999), and who were in this situation for at least two of the years 2000–2008 (1990–1998). The long-term poor (coded 1) are contrasted to the non-poor (coded 0), excluding the short-term poor (coded missing) in order to distinguish whether long-term poverty is particularly detrimental (as compared to absolute poverty in general).
- *Relative poverty* is defined, according to the EU standard, as having a disposable equivalized income that is lower than 60 % of the median income in Sweden the year in question (EU 2005).^4^ As for absolute poverty, this variable is based on incomes the year prior to the survey year. Self-employed are excluded.

### Social and Participation Outcomes

#### Primary (core) Social Relations

- *Social support* The value 1 (has support) is given to those who have answered in the positive to three questions about whether one has a close friend who can help if one (a) gets sick, (b) needs someone to talk to about troubles, or (c) needs company. Those who lack support in at least one of these respects are coded 0 (lack of support).
- *Frequent social relations* This variable is based on four questions about how often one meets (a) relatives and (b) friends, either (i) at ones’ home or (ii) at the home of those one meets, with the response set being “yes, often”, “sometimes”, and “no, never”. Respondents are defined as having frequent relations (1) if they have at least one “often” of the four possible and no “never”,^5^ and 0 otherwise.

#### Secondary (fringe) Social Relations/Participation

- *Political participation*: Coded 1 (yes) if one during the last 12 months actively participated (held an elected position or was at a meeting) in a trade union or a political party, and 0 (no) otherwise.^6^
- *Organizational activity*: Coded 1 (yes) if one is a member of an organization and actively participate in its activities at least once in a year, and 0 (no) otherwise.

### Control Variables

- Sex
- Age (in years)
- *Educational qualifications* in 2010 (five levels according to a standard schema used by Statistics Sweden (1985), entered as dummy variables)
- *Civil status* distinguishes between single and cohabiting/married persons, and is used as a time-varying covariate (TVC) where we register any changes from couple to single and vice versa.
- *Immigrant origin* is coded 1 if both parents were born in any country outside Sweden, 0 otherwise.
- *Labour market status* is also used as a TVC, with four values indicating labour market participation (yes/no) in 2000 and 2010, respectively.
- *Global self*-*rated health* in 2000, with three response alternatives: Good, bad, or in between.^7^

Table 1 shows descriptive statistics for the 2 years we study, 2000 and 2010 (percentages in the upper panel; averages, standard deviations, max and min values in the lower panel). Recall that the sample is longitudinal with the same respondents appearing in both years. This means, naturally, that the sample ages 10 years between the waves, the upper age limit being pushed up from 65 to 75. Both the change over years and the ageing of the sample have repercussions for their conditions: somewhat more have poor health, for example, fewer lack social support but more lack frequent social relations, and more are single in 2010 (where widows are a growing category). The group has however improved their economic conditions, with a sizeable reduction in poverty rates. Most of the changes are in fact period effects, and it is particularly obvious for the change in poverty—in 2000 people still suffered from the deep recession in Sweden that begun in 1991 and started to turn in 1996/97 (Jonsson et al. 2010), while the most recent international recession (starting in 2008/09) did not affect Sweden that much.

**Table 1:** Descriptive statistics of dependent and independent variables in the LNU panel

Categorical variables	% in 2000	% in 2010	N
Social support	93	95	3150
Frequent social relations	89	84	3157
Civic participation (organizations)	52	44	3139
Political participation	27	24	3157
Economically deprived	15	10	3083
Poor (“absolute”)	15	6	3156
Poor (relative)	19	10	3139
Long-term poor/social assistance	12	5	3156
Deprived + income-poor/social assistance	7	3	3082
Unemployed	5	3	3153
Woman	49		3157
Single	25	29	3157
Immigrant origin	11		3157
*Education*			3149
Comprehensive school		15	
Vocational secondary		28	
Academic upper secondary		17	
Short-cycle tertiary		16	
University degree		24	
*Health*			3157
Good	78	75	
In between	18	20	
Poor	4	5	

Metric variable	Mean	Stddev	Min	Max	N
Age 2010	52	13	29	75	3157

N for variables used as change variables pertains to non-missing observations in both 2000 and 2010

The overall decrease in poverty masks changes that our respondents experienced between 2000 and 2010: Table 2 reveals these for the measure of economic deprivation, showing the outflow (row) percentages and the total percentages (and the number of respondents in parentheses). It is evident that there was quite a lot of mobility out of poverty between the years (61 % left), but also a very strong relative risk of being found in poverty in 2010 among those who were poor in 2000 (39 vs. 5 % of those who were non-poor in 2000). Of all our respondents, the most common situation was to be non-poor both years (81 %), while few were poor on both occasions (6 %). Table 2 also demonstrates some small cell numbers: 13.3 % of the panel (9.4 % + 3.9 %), or a good 400 cases, changed poverty status, and these cases are crucial for identifying our models. As in many panel studies based on survey data, this will inevitably lead to some problems with large standard errors and difficulties in arriving at statistically significant and precise estimates; but to preview the findings, our results are surprisingly consistent all the same.

**Table 2:** Mobility in poverty (measured as economic deprivation) in Sweden between 2000 and 2010

	Poor in 2010	Not poor in 2010	Total
*Poor in 2000*			
Row %	39.1	60.9	100.0
Total %	6.0	9.4	15.4
(n)	(186)	(290)	(476)
*Not poor in 2000*			
Row %	4.6	95.4	100.0
Total %	3.9	80.7	84.6
(n)	(119)	(2488)	(2607)
*Total*	9.9	90.1	100.0
(n)	(305)	(2778)	(3083)

Outflow percentage (row %), total percentage, and number of cases (in parentheses). LNU panel 2000–2010

## Results

**Fig. 1:**
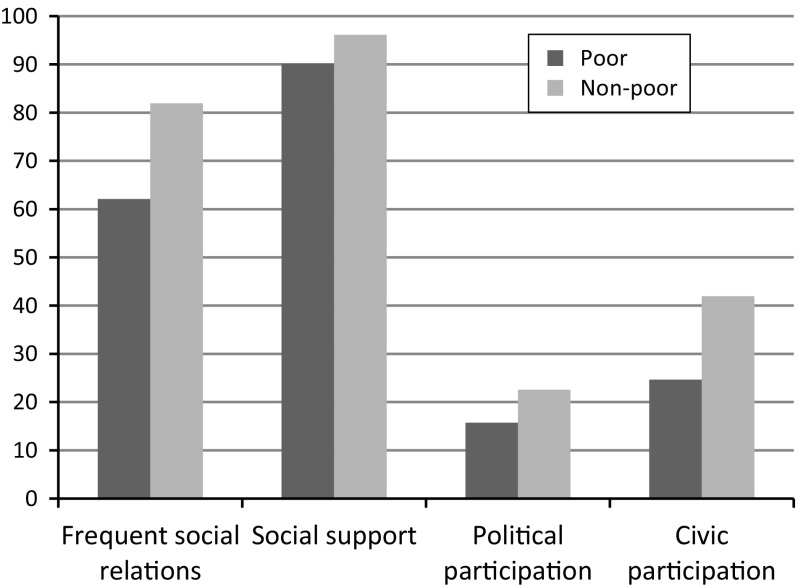
The relation between poverty (measured as economic deprivation) and social relations/participation in Sweden, LNU 2010. N = 5271

We begin with showing descriptive results of how poverty is associated with our outcome variables, using the economic deprivation measure of poverty.^8^ Figure 1 confirms that those who are poor have worse social relationships and participate less in political life and in organizations. Poverty is thus connected with both primary and secondary social relations.

The descriptive picture in Fig. 1 does not tell us anything about the causal nature of the relation between poverty and social outcomes, only that such a relation exists, and that it is in the predicted direction: poor people have weaker social relations, less support, and lower levels of political and civic participation. Our task now is to apply more stringent statistical models to test whether the relation we have uncovered is likely to be of a causal nature. This means that we must try to rid the association of both the risk for reverse causality—that, for example, a weaker social network leads to poverty—and the risk that there is a common underlying cause of both poverty and social outcomes, such as poor health or singlehood.

### The Change Model

First, as we have panel data, we can study the difference in change across two time-points T (called t_0_ and t_1_, respectively) in an outcome variable (e.g., social relations), between groups (i.e. those who changed poverty status versus those who did not). The respondents are assigned to either of these groups on the grounds of entering or leaving poverty; in the first case, one group is non-poor at t_0_ but experiences poverty at t_1_, and the change in this group is compared to the group consisting of those who are non-poor both at t_0_ and t_1_. The question in focus then is: Do social relations in the group entering poverty worsen in relation to the corresponding change in social relations in the group who remains non-poor? Because we have symmetric hypotheses of the effect of poverty on social outcomes—assuming leaving poverty has positive consequences similar to the negative consequences of entering poverty—we also study whether those who exit poverty improve their social outcomes as compared to those remaining poor. We ask, that is, not only what damage falling into poverty might have for social outcomes, but also what “social gains” could be expected for someone who climbs out of poverty.

Thus, in our analyses we use two different “change groups”, *poverty leavers* and *poverty entrants*, and two “comparison groups”, *constantly poor* and *never poor*, respectively.^9^ The setup comparing the change in social outcomes for those who change poverty status and those who do not is analogous to a so-called difference-in-difference design, but as the allocation of respondents to comparison groups and change groups in our data cannot be assumed to be random (as with control groups and treatment groups in experimental designs), we take further measures to approach causal interpretations.

### Accounting for the Starting Value of the Dependent Variable

An important indication of the non-randomness of the allocation to the change and comparison groups is that their average values of the social outcomes (i.e. the dependent variable) at t_0_ differ systematically: Those who become poor between 2000 and 2010 have on average worse social outcomes already in 2000 than those who stay out of poverty. Similarly, those who stay in poverty both years have on average worse social outcomes than those who have exited poverty in 2010. In order to further reduce the impact of unobserved variables, we therefore make all comparisons of changes in social outcomes between t_0_ and t_1_ for fixed t_0_ values of *both* social outcome and poverty status.

As we use dichotomous outcome variables, we get eight combinations of poverty and outcome states (2 × 2 × 2 = 8), and four direct strategic comparisons:

1. *Poverty leavers versus constantly poor, positive social outcome in 2000*, showing if those who exit poverty have a higher chance of maintaining the positive social outcome than those who stay in poverty
2. *Poverty leavers versus constantly poor, negative social outcome in 2000*, showing if those who exit poverty have a higher chance of improvement in the social outcome than those who stay in poverty
3. *Poverty entrants versus never poor, positive social outcome in 2000*, showing if those who enter poverty have a higher risk of deterioration in the social outcome than those who stay out of poverty, and
4. *Poverty entrants versus never poor, negative social outcome in 2000*, showing if those who enter poverty have a lower chance of improvement in the social outcome.

Thus, we hold the initial social situation and poverty status fixed, letting only the poverty in 2010 vary.^10^ The analytical strategy is set out in Table 3, showing estimates of the probability to have frequent social relations in 2010, for poverty defined (as in Table 2 and Fig. 1 above) as economic deprivation.

**Table 3:** Per cent with frequent social relations in “comparison” and “change” groups in 2000 and 2010, according to initial value on social relations in 2000 and poverty (measured as economic deprivation) in 2000 and 2010

	Non-frequent social relations 2000	Frequent social relations 2000
*Economic deprivation*	*Proportion with frequent social relations 2010*	
0–0 (never poor)	0.59	0.90
0–1 (became poor)	0.52	0.72
*Difference*	−0.07	−0.17
1–1 (constantly poor)	0.39	0.72
1–0 (escaped poverty)	0.72	0.86
*Difference*	0.33	0.14

LNU panel 2000–2010. N = 3083

The figures in Table 3 should be read like this: 0.59 in the upper left cell means that among those who were poor neither in 2000 nor in 2010 (“never poor”, or 0–0), and who had non-frequent social relations to begin with, 59 % had frequent social relations in 2010. Among those never poor who instead started out with more frequent social relations, 90 per cent had frequent social relations in 2010. This difference (59 vs. 90) tells us either that the initial conditions were important (weak social relations can be inherently difficult to improve) or that there is heterogeneity within the group of never poor people, such as some having (to us perhaps unobserved) characteristics that support relation building while others have not.

Because our strategy is to condition on the initial situation in order to minimize the impact of initial conditions and unobserved heterogeneity, we focus on the comparisons across columns. If we follow each column downwards, that is, for a given initial social outcome (weak or not weak social relations, respectively) it is apparent that the outcome is worse for the “poverty entrants” in comparison with the “never poor” (upper three lines). Comparing the change group [those who became poor (0–1)] with the comparison group [never poor (0–0)] for those who started out with weak social relations (left column), the estimated probability of frequent social relations in 2010 is 7 % points lower for those who became poor. Among those who started out with frequent relations, those who became poor have a 17 % points lower probability of frequent relations in 2010 than those who stayed out of poverty.

If we move down Table 3, to the three bottom lines, the change and comparison groups are now different. The comparison group is the “constantly poor” (1–1), and the change group are “poverty leavers” (1–0). Again following the columns downwards, we can see that the change group improved their social relations in comparison with the constantly poor; and this is true whether they started out with weak social relations or not. In fact, the chance of improvement for those who started off with non-frequent social relations is the most noteworthy, being 33 % units higher for those who escaped poverty than for those who did not. In sum, Table 3 suggests that becoming poor appears to be bad for social relations whereas escaping poverty is beneficial.

### Expanding the Model

The model exemplified in Table 3 is a panel model that studies change across time within the same individuals, conditioning on their initial state. It does away with time-constant effects of observed and unobserved respondent characteristics, and although this is far superior to a cross-sectional model (such as the one underlying Fig. 1) there are still threats to causal interpretations. It is possible (if probably unusual) that permanent characteristics may trigger a *change* over time in both the dependent and independent variables; or, put in another way, whether a person stays in or exits poverty may be partly caused by a variable that also predicts change in the outcome (what is sometimes referred to as a violation of the “common trend assumption”). In our case, we can for example imagine that health problems in 2000 can affect who becomes poor in 2010, at t_1_, and that the same health problems can lead to a deterioration of social relations between 2000 and 2010, so even conditioning on the social relations at t_0_ will not be enough. This we handle by adding control variables, attempting to condition the comparison of poor and non-poor also on sex, age, highest level of education (in 2010), immigrant status, and health (in 2000).^11^

Given the set-up of our data—with 10 years between the two data-points and with no information on the precise time ordering of poverty and social outcomes at t_1_, the model can be further improved by including change in some of the control variables. It is possible, for example, that a non-poor and married respondent in 2000 divorced before 2010, triggering both poverty and reduced social relations at the time of the interview in 2010.^12^ There are two major events that in this way may bias our results, divorce/separation and unemployment (because each can lead to poverty, and possibly also affect social outcomes). We handle this by controlling for variables combining civil status and unemployment in 2000 as well as in 2010. To the extent that these factors are a consequence of becoming poor, there is a risk of biasing our estimates downwards (e.g., if becoming poor increases the risk of divorce). However, as there is no way to distinguish empirically whether control variables (divorce, unemployment) or poverty changed first we prefer to report conservative estimates.^13^

Throughout, we use logistic regression to estimate our models (one model for each social outcome and poverty definition). We create a dummy variable for each of the combinations of poverty in 2000, poverty in 2010 and the social outcome in 2000, and alternate the reference category in order to get the four strategic comparisons described above. Coefficients do thus express the distance between the relevant change and comparison groups. The coefficients reported are average marginal effects (AME) for a one-unit change in the respective poverty variable (i.e. going from non-poor to poor and vice versa), which are straightforwardly interpretable as percentage unit differences and (unlike odds ratios or log odds ratios) comparable across models and outcomes (Mood 2010).

### Regression Results

As detailed above, we use changes over time in poverty and social outcomes to estimate the effects of interest. The effect of poverty is allowed to be heterogeneous, and is assessed through four comparisons of the social outcome in 2010 (Y_1_):

1. Those entering poverty relative to those in constant non-poverty (P_01_ = 0,1 vs. P_01_ = 0,0) when both have favourable social outcomes at t_0_ (Y_0_ = 1)
2. Those exiting poverty relative to those in constant poverty (P_01_ = 1,0 vs. P_01_ = 1,1) when both have favourable social outcomes at t_0_ (Y_0_ = 1)
3. Those entering poverty relative to those in constant non-poverty (P_01_ = 0,1 vs. P_01_ = 0,0) when both have non-favourable social outcomes at t_0_ (Y_0_ = 0)
4. Those exiting poverty relative to those in constant poverty (P_01_ = 1,0 vs. P_01_ = 1,1) when both have non-favourable social outcomes at t_0_ (Y_0_ = 0)

Poverty is a rare outcome, and as noted above it is particularly uncommon to enter poverty between 2000 and 2010 because of the improving macro-economic situation. Some of the social outcomes were also rare in 2000. This unfortunately means that in some comparisons we have cell frequencies that are prohibitively small, and we have chosen to exclude all comparisons involving cells where N < 20.

The regression results are displayed in Table 4. To understand how the estimates come to be, consider the four in the upper left part of the Table (0.330, 0.138, −0.175 and −0.065), reflecting the effect of poverty, measured as economic deprivation, on the probability of having frequent social relations. Because these estimates are all derived from a regression without any controls, they are identical (apart from using three decimal places) to the percentage comparisons in Table 3 (0.33, 0.14, −0.17, −0.07), and can be straightforwardly interpreted as average differences in the probability of the outcome in question. From Table 4 it is clear that the three first differences are all statistically significant, whereas the estimate −0.07 is not (primarily because those who entered poverty in 2010 and had infrequent social relations in 2000 is a small group, N = 25).

**Table 4:** Average marginal effects (from logistic regression) of five types of poverty (1–5) on four social outcomes (A-D) comparing those with different poverty statuses in 2000 and 2010 and conditioning on the starting value of the social outcome (in 2000)

	Economically deprived (1)		Absolute poor (2)		Deprived and abs. poor (3)		Long-term poor (4)		Relative poor (5)	
	No controls	Controls	No controls	Controls	No controls	Controls	No controls	Controls	No controls	Controls
*Social relations (A)*										
P11 versus P10, Y0 = negative	**0.330**	**0.210**	**0.354**	0.172	**0.432**	0.291	**0.296**	0.134	0.082	0.130
	(0.000)	(0.029)	(0.000)	(0.114)	(0.000)	(0.052)	(0.008)	(0.251)	(0.479)	(0.240)
P11 versus P10, Y0 = positive	**0.138**	**0.081**	**0.154**	0.050	0.035	−0.048	**0.197**	0.065	0.026	0.034
	(0.002)	−0.048	−0.005	(0.260)	(0.676)	(0.374)	(0.003)	(0.225)	(0.546)	(0.455)
P00 versus P01, Y0 = positive	−**0.175**	−**0.135**	−**0.122**	−0.070	−**0.287**	−**0.188**	−**0.156**	−0.091	0.013	−0.013
	(0.000)	(0.002)	(0.009)	(0.084)	(0.001)	(0.012)	(0.012)	(0.082)	(0.583)	(0.645)
P00 versus P01, Y0 = negative	−0.065	−0.048	–	–	–	–	–	–	0.116	0.042
	(0.536)	(0.635)							(0.241)	(0.668)
*Social support (B)*										
P11 versus P10, Y0 = negative	**0.233**	0.102	0.200	0.102	–	–	0.200	0.108	–	–
	(0.030)	(0.190)	(0.079)	(0.177)			(0.133)	(0.235)		
P11 versus P10, Y0 = positive	0.030	0.002	**0.082**	0.018	0.056	−0.006	**0.100**	0.021	0.042	0.052
	(0.248)	(0.928)	−0.039	(0.532)	(0.356)	(0.882)	(0.039)	(0.524)	(0.147)	(0.105)
P00 versus P01, Y0 = positive	−**0.066**	−**0.050**	−**0.059**	−0.045	−**0.151**	−**0.128**	−0.063	−0.045	**0.026**	**0.024**
	(0.023)	(0.050)	(0.050)	(0.089)	(0.025)	(0.037)	(0.112)	(0.176)	(0.002)	(0.022)
P00 versus P01, Y0 = negative	–	–	–	–	–	–	–	–	–	–
*Political participation (C)*										
P11 versus P10, Y0 = negative	**0.119**	**0.109**	**0.105**	**0.083**	0.047	0.032	**0.110**	**0.091**	**0.091**	**0.081**
	(0.001)	(0.006)	(0.003)	(0.038)	(0.391)	(0.616)	(0.005)	(0.041)	(0.015)	−0.034
P11 versus P10, Y0 = positive	–	–	–	–	–	–	–	–	–	–
P00 versus P01, Y0 = negative	−**0.082**	−**0.074**	−**0.077**	−0.066	−0.077	−0.058	−0.044	−0.034	−0.044	−0.036
	(0.008)	(0.023)	(0.029)	(0.090)	(0.140)	(0.343)	(0.374)	(0.516)	(0.113)	(0.222)
P00 versus P01, Y0 = positive	−0.0508	−0.023	0.111	0.104	–	–	–	–	−0.121	−0.121
	(0.589)	(0.815)	(0.301)	(0.334)					(0.113)	(0.115)
*Organizational activity (D)*										
P11 versus P10, Y0 = negative	**0.100**	0.091	0.048	0.029	0.093	0.108	0.089	0.083	0.026	0.012
	(0.032)	(0.091)	(0.408)	(0.680)	(0.155)	(0.188)	(0.164)	(0.295)	(0.636)	(0.845)
P11 versus P10, Y0 = positive	0.068	0.047	**0.190**	0.188	–	–	0.149	0.151	−0.017	−0.067
	(0.372)	(0.543)	(0.041)	(0.055)			(0.157)	(0.167)	(0.843)	(0.396)
P00 versus P01, Y0 = negative	−0.078	−0.039	−**0.182**	−**0.172**	−**0.198**	−**0.180**	−**0.173**	−**0.156**	0.009	0.029
	(0.126)	(0.493)	(0.000)	(0.001)	(0.008)	(0.042)	(0.003)	(0.017)	(0.853)	(0.570)
P00 versus P01, Y0 = positive	−**0.160**	−0.125	−0.008	0.032	−0.080	−0.056	−0.008	0.054	−0.039	0.002
	(0.035)	(0.107)	(0.920)	(0.682)	(0.478)	(0.625)	(0.943)	(0.611)	(0.453)	(0.973)

Right columns control for sex, education, age, immigrant status, health in 2000, civil status change between 2000 and 2010, and unemployment change between 2000 and 2010. *P* values in parentheses. Excluded estimates involve variable categories with N < 20. Shaded cells are in hypothesized direction, bold estimates are statistically significant (*P* < 0.05). N in regressions: 1A: 3075; 1B: 3073; 1C: 3075; 1D: 3069; 2A: 3144; 2B: 3137; 2C: 3144; 2D: 3130; 3A: 3074, 3B: 3072; 3C: 3074; 3D: 3068; 4A: 2995; 4B: 2988; 4C: 2995; 4D: 2981; 5A: 3128; 5B: 3121; 5C: 3128; 5D: 3114

In the column to the right, we can see what difference the controls make: the estimates are reduced, but not substantially so, and the three first differences are still statistically significant.

The estimates for each social outcome, reflecting the four comparisons described above, support the hypothesis of poverty affecting social relations negatively (note that the signs of the estimates should differ in order to do so, the upper two being positive as they reflect an effect of the exit from poverty, and the lower two being negative as they reflect an effect of entering poverty). We have indicated support for the hypothesis in Table 4 by shading the estimates and standard errors for estimates that go in the predicted direction.

Following the first two columns down, we can see that there is mostly support for the hypothesis of a negative effect of poverty, but when controlling for other variables, the effects on social support are not impressive. In fact, if we concentrate on each social outcome (i.e., row-wise), one conclusion is that, when controlling for confounders, there are rather small effects of poverty on the probability of having access to social support. The opposite is true for political participation, where the consistency in the estimated effects of poverty is striking.

If we instead follow the columns, we ask whether any of the definitions of poverty is a better predictor of social outcomes than the others. The measure of economic deprivation appears to be the most stable one, followed by absolute poverty and the combined deprivation/absolute poverty variable.^14^ The relative poverty measure is less able to predict social outcomes: in many instances it even has the non-expected sign. Interestingly, long-term poverty (as measured here) does not appear to have more severe negative consequences than absolute poverty in general.

Because some of our comparison groups are small, it is difficult to get high precision in the estimates, efficiency being a concern particularly in view of the set of control variables in Table 4. Only 14 out of 62 estimates in models with controls are significant and in the right direction. Nonetheless, with 52 out of 62 estimates in these models having the expected sign, we believe that the hypothesis of a negative effect of poverty on social outcomes receives quite strong support.

Although control variables are not shown in the table, one thing should be noted about them: The reduction of coefficients when including control variables is almost exclusively driven by changes in civil status.^15^ The time constant characteristics that are included are cross-sectionally related to both poverty and social outcomes, but they have only minor impacts on the estimated effects of poverty. This suggests that the conditioning on prior values of the dependent and independent variables eliminates much time invariant heterogeneity, which increases the credibility of estimates.

## Conclusions

We set out to test a fundamental, but rarely questioned assumption in dominating definitions of poverty: whether shortage of economic resources has negative consequences for social relations and participation. By using longitudinal data from the Swedish Level-of-living Surveys 2000 and 2010, including repeated measures of poverty (according to several commonly used definitions) and four social outcome variables, we are able to come further than previous studies in estimating the relation between poverty and social outcomes: Our main conclusion is that there appears to be a causal relation between them.

Panel models suggest that falling into poverty increases the risk of weakening social relations and decreasing (civic and political) participation. Climbing out of poverty tends to have the opposite effects, a result that strengthens the interpretation of causality. The sample is too small to estimate the effect sizes with any precision, yet they appear to be substantial, with statistically significant estimates ranging between 5 and 21 % units.

While these findings are disquieting insofar as poverty goes, our results also suggest two more positive results. First, the negative effects of poverty appear to be reversible: once the private economy recovers, social outcomes improve. Secondly, the negative consequences are less for the closest social relations, whether there is someone there in cases of need (sickness, personal problems, etc.). This is in line with an interpretation of such close relations being unconditional: our nearest and dearest tend to hang on to us also in times of financial troubles, which may bolster risks for social isolation and psychological ill-being,

Our finding of negative effects of poverty on civic and political participation relates to the fears of a “downward spiral of social exclusion”, as there is a risk that the loss of less intimate social relations shrinks social networks and decreases the available social capital in terms of contacts that can be important for outcomes such as finding a job (e.g., Lin 2001; Granovetter 1974). However, Gallie et al. (2003) found no evidence for any strong impact of social isolation on unemployment, suggesting that the negative effects on social outcomes that we observe are unlikely to lead to self-reinforcement of poverty. Nevertheless, social relations are of course important outcomes in their own right, so if they are negatively affected by poverty it matters regardless of whether social relations in turn are important for other outcomes. Effects on political and civic participation are also relevant in themselves beyond individuals’ wellbeing, as they suggest a potentially democratic problem where poor have less of a voice and less influence on society than others.

Our results show the merits of our approach, to study the relation between poverty and social outcomes longitudinally. The fact that the poor have worse social relations and lower participation is partly because of selection. This may be because the socially isolated, or those with a weaker social network, more easily fall into poverty; or it can be because of a common denominator, such as poor health or social problems. But once we have stripped the analysis of such selection effects, we also find what is likely to be a causal relation between poverty and social relations. However, this effect of poverty on social outcomes, in turn, varies between different definitions of poverty. Here it appears that economic deprivation, primarily indicated by the ability of raising money with short notice, is the strongest predictor of social outcomes. Income poverty, whether in absolute or (particularly) relative terms, are weaker predictors of social outcomes, which is interesting as they are the two most common indicators of poverty in existing research.

Even if we are fortunate to have panel data at our disposal, there are limitations in our analyses that render our conclusions tentative. One is that we do not have a random allocation to the comparison groups at t_0_; another that there is a 10-year span between the waves that we analyze, and both poverty and social outcomes may vary across this time-span. We have been able to address these problems by conditioning on the outcome at t_0_ and by controlling for confounders, but in order to perform more rigorous tests future research would benefit from data with a more detailed temporal structure, and preferably with an experimental or at least quasi-experimental design.

Finally, our analyses concern Sweden, and given the position as an active welfare state with a low degree of inequality and low poverty rates, one can ask whether the results are valid also for other comparable countries. While both the level of poverty and the pattern of social relations differ between countries (for policy or cultural reasons), we believe that the mechanisms linking poverty and social outcomes are of a quite general kind, especially as the “costs for social participation” can be expected to be relative to the general wealth of a country—however, until comparative longitudinal data become available, this must remain a hypothesis for future research.

## Footnotes

1 http://www.sofi.su.se/english/2.17851/research/three-research-departments/lnu-level-of-living.

2 We have tested various alternative codings and the overall pattern of results in terms of e.g., direction of effects and differences across poverty definitions are similar, but more difficult to present in an accessible way.

3 Our deprivation questions are however designed to reduce the impact of subjectivity by asking, e.g., about getting a specified sum within a specified time (see below).

4 In the equivalence scale, the first adult gets a weight of one, the second of 0.6, and each child gets a weight of 0.5.

5 We have also tried using single indicators (either a/b or i/ii) without detecting any meaningful difference between them. One would perhaps have assumed that poverty would be more consequential for having others over to one’s own place, but the absence of support for this can perhaps be understood in light of the strong social norm of reciprocity in social relations.

6 We have refrained from using information on voting and membership in trade unions and political parties, because these indicators do not capture the active, social nature of civic engagement to the same extent as participation in meetings and the holding of positions.

7 We have also estimated models with a more extensive health variable, a s*ymptom index*, which sums responses to 47 questions about self-reported health symptoms. However, this variable has virtually zero effects once global self-rated health is controlled, and does not lead to any substantive differences in other estimates. Adding the global health measure and the symptom index as TVC had no effect either.

8 Using the other indicators of poverty yields very similar results, although for some of those the difference between poor and non-poor is smaller.

9 We call these comparison groups ”never poor” and ”constantly poor” for expository purposes, although their poverty status pertains only to the years 2000 and 2010, i.e., without information on the years in between.

10 With this design we allow different effects of poverty on improvement versus deterioration of the social outcome. We have also estimated models with a lagged dependent variable, which constrains the effects of poverty changes to be of the same size for deterioration as for improvement of the social outcome. Conclusions from that analysis are roughly a weighted average of the estimates for deterioration and improvement that we report. As our analyses suggest that effects of poverty differ in size depending on the value of the lagged dependent variable (the social outcome) our current specification gives a more adequate representation of the process.

11 We have also tested models with a wider range of controls for, e.g., economic and social background (i.e. characteristics of the respondent’s parents), geography, detailed family type and a more detailed health variable, but none of these had any impact on the estimated poverty effects.

12 It is also possible that we register reverse causality, namely if worsening social outcomes that occur after t_0_ lead to poverty at t_1_. This situation is almost inevitable when using panel data with no clear temporal ordering of events occurring between waves. However, reverse causality strikes us, in this case, as theoretically implausible.

13 We have also estimated models controlling for changes in health, which did not change the results.

14 If respondents’ judgments of the deprivation questions (access to cash margin and ability to pay rent, food, bills etc.) change due to non-economic factors that are related to changes in social relations, the better predictive capacity of the deprivation measure may be caused by a larger bias in this measure than in the (register-based) income measures.

15 As mentioned above, this variable may to some extent be endogenous (i.e., a mediator of the poverty effect rather than a confounder), in which case we get a downward bias of estimates.

## References

[CR1] Atkinson AB, Cantillon B, Marlier E, Nolan B (2002). Social indicators: The EU and social inclusion.

[CR2] Attree P (2006). The social costs of child poverty: A systematic review of the qualitative evidence. Children and Society.

[CR3] Bane MJ, Ellwood DT (1986). Slipping into and out of Poverty: The Dynamics of Spells. Journal of Human Resources.

[CR4] Barnes M, Heady C, Middleton S, Millar J, Papadopoulos F, Room G, Tsakloglou P (2002). Poverty and social exclusion in Europe.

[CR5] Böhnke P (2008). Are the poor socially integrated? The link between poverty and social support in different welfare regimes. Journal of European Social Policy.

[CR6] Bradshaw J, Finch N (2003). Overlaps in dimensions of poverty. Journal of Social Policy.

[CR7] Callan T, Nolan B, Whelan CT (1993). Resources, deprivation, and the measurement of poverty. Journal of Social Policy.

[CR8] Corak M (2006). Principles and practicalities for measuring child poverty in the rich countries. International Social Security Review.

[CR9] Dahl E, Flotten T, Lorentzen T (2008). Poverty dynamics and social exclusion: An analysis of Norwegian panel data. Journal of Social Policy.

[CR10] Duncan GJ, Gustafsson B, Hauser R, Schmauss G, Messinger H, Muffels R, Nolan B, Ray J-C (1993). Poverty dynamics in eight countries. Journal of Population Economics.

[CR11] Duncan GJ, Rodgers W (1991). Has children’s poverty become more persistent?. American Sociological Review.

[CR12] Galbraith J (1958). The affluent society.

[CR13] Gallie D, Paugam S, Jacobs S (2003). Unemployment, poverty and social isolation: Is there a vicious cycle of social exclusion?. European Societies.

[CR14] Gordon D, Adelman L, Ashworth K, Bradshaw J, Levitas R, Middleton S, Pantazis C, Patsios D, Payne S, Townsend P, Williams J (2000). Poverty and social exclusion in Britain.

[CR15] Gore C, Rodgers G, Gore C, Figueiredo JB (1995). Introduction: Markets, citizenship and social exclusion. Social exclusion: Rhetoric, reality, responses.

[CR16] Granovetter, M. S. (1974). *Getting a job. A study of contacts and careers*. Cambridge: Harvard University Press.

[CR17] Halleröd B (1995). The truly poor: Direct and indirect measurement of consensual poverty in Sweden. Journal of European Social Policy.

[CR18] Hills J, Le Grand J, Piachaud D (2002). Understanding social exclusion.

[CR19] Jansson, K. (2000). Inkomstfördelningen under 1990-talet. In *Välfärd och försörjning* 2000, pp. 15–60. SOU 2000:40.

[CR20] Jonsson, J. O., Mood, C., & Bihagen, E. (2010). Fattigdomens förändring, utbredning och dynamik, Chapter 3. In *Social Rapport 2010*. Stockholm: Socialstyrelsen.

[CR21] Jonsson, J. O., & Östberg, V. (2004). Resurser och levnadsförhållanden bland ekonomiskt utsatta 10-18-åringar: Analys av Barn-LNU och Barn-ULF. pp. 203–55 in *Ekonomiskt utsatta barn,* Socialdepartementet, Ds. 2004:41. Stockholm: Fritzes.

[CR22] Levitas R, Pantazis C, Gordon D, Levitas R (2006). The concept and measurement of social exclusion. Poverty and social exclusion in Britain.

[CR23] Lin, N. (2001). *Social capital. A theory of social structure and action*. Cambridge: Cambridge University Press.

[CR24] Mack J, Lansley S (1985). Poor Britain.

[CR25] Mood C (2010). Logistic regression: Why we cannot do what we think we can do and what we can do about it. European Sociological Review.

[CR26] Nolan B, Whelan CT (2011). Poverty and deprivation in Europe.

[CR27] OECD (2008). Growing unequal? Income distribution and poverty in OECD countries.

[CR28] Paugam S, Room G (1995). The spiral of precariousness: A multidimensional approach to the process of social disqualification in France. Beyond the threshold: The measurement and analysis of social exclusion.

[CR29] Ridge T, Millar J (2011). Following families: Working lone-mother families and their children. Social Policy & Administration.

[CR30] Ringen S (1988). Direct and indirect measures of poverty. Journal of Social Policy.

[CR31] Rodgers G, Gore C, Figueiredo JB (1995). Social exclusion: Rhetoric, reality, responses.

[CR32] Rodgers JR, Rodgers JL (1993). Chronic poverty in the United States. Journal of Human Resources.

[CR33] Room G (1995). Beyond the threshold: The measurement and analysis of social exclusion.

[CR34] Sen A (1983). Poor, relatively speaking. Oxford Economic Papers.

[CR35] Smith, A. (1776). *An inquiry into the nature and causes of the wealth of nations* (republished by R. H. Campbell and A. S. Skinner (Eds.). Oxford: Clarendon Press, 1976).

[CR36] Townsend P (1979). Poverty in the United Kingdom.

[CR37] van den Bosch K (2001). Identifying the poor: Using subjective and consensual measures.

[CR38] United Nations. (1995). *United nations world summit (Copenhagen) for social development. programme of action*, Chapter 2. New York: United Nations.

[CR39] UNICEF. (2012). Measuring child poverty. New league tables of child poverty in the world’s rich countries. In *Innocenti Report Card 10*. Florence: UNICEF Innocenti Research Centre.

[CR40] Veblen T (1899). The theory of the leisure class.

[CR41] Whelan CT, Layte R, Maitre B (2003). Persistent income poverty and deprivation in the European Union: An analysis of the first three waves of the European community household panel. Journal of Social Policy.

